# GP strategies to avoid imaging overuse. A qualitative study in Norwegian general practice

**DOI:** 10.1080/02813432.2022.2036480

**Published:** 2022-02-21

**Authors:** Karina Ellingsen Walderhaug, Marie Kaltenborn Nyquist, Bente Prytz Mjølstad

**Affiliations:** a NTNU, Norwegian University of Science and Technology, Trondheim, Norway; b General Practice Research Unit, Department of Public Health and Nursing, NTNU, Norwegian University of Science and Technology, Trondheim, Norway

**Keywords:** Primary Health Care, General Practice, Physician-Patient Relations, Diagnostic Imaging, Communication, Medical Overuse

## Abstract

**Objectives:**

The aim of the study was to identify general practitioners’ (GPs) strategies to avoid unnecessary diagnostic imaging when encountering patients with such expectations and to explore how patients experience these strategies.

**Design, setting and subjects:**

We conducted a qualitative study that combined observations of consultations and interviews with GPs and patients. A total of 24 patients visiting nine different GPs in two Norwegian urban areas were included in the study. Of these, 12 consultations were considered suitable for studying GP strategies and were therefore selected for a more thorough analysis.

**Main outcome measures:**

GPs’ communication strategies to avoid unnecessary medical imaging and patients’ experiences with such strategies.

**Results:**

Five categories of strategies were identified: (1) wait and see – or suggest an alternative; (2) the art of rejection; (3) seek support from a professional authority; (4) partnership and shared decision-making and (5) reassurance, normalisation and recognition. The GPs often used multiple strategies. Factors related to a long-term doctor–patient relationship seemed to influence both communication and how both parties experienced the decision. Three important factors were evident: the patient trusted the doctor, the doctor knew the patient’s medical history and the doctor knew the patient as a person. The patients seemed to be generally satisfied with the outcomes of the consultations.

**Conclusion:**

GPs largely combine different strategies when meeting patients’ expectations of diagnostic imaging that are not strictly medically indicated. Continuity of the doctor–patient relationship with good personal knowledge and trust between doctor and patient appeared crucial for patients to accept the doctors' decisions.

**Key points**
- GPs usually combine a broad range of strategies to avoid unnecessary medical imaging
- The patients appeared generally satisfied regardless of the strategy the strategy used by the GPs and even where their referral request were rejected
- Factors related to a long-term doctor–patient relationship appeared decisive

## Introduction

*Primum non nocere* – first, do no harm – derives from Hippocrates and represents a fundamental ethical principle in medicine. It has gained momentum in recent decades in line with the growing awareness of medical overactivity. This issue has been highlighted internationally through a series of articles such as ‘Too Much Medicine’ in *BMJ* [1] and ‘Less Is More’ in *JAMA* [2], as well as in several campaigns. Best known is the widespread ‘Choosing Wisely’ campaign, which originated in the United States, and the Norwegian Medical Association’s equivalent ‘Gjør kloke valg’ [Make wise choices], was launched in 2018 [3].

The definitions of terms related to medical overactivity are often ambiguous. In general, healthcare overuse consists of both unnecessary testing, overdiagnosis and overtreatment. Brodersen et al. described overdiagnosis [4] as ‘making people patients unnecessarily, by identifying problems that were never going to cause harm or by medicalising ordinary life experiences through expanded definitions of diseases’, in turn causing the patient more harm than good. The risk of overdiagnosis increases proportionally with the extent of overtesting [4].

The harm of medical overactivity is difficult to communicate to the public. Not only can medical examinations expose the patient to risk, but unnecessary treatment can also cause side effects and complications as well as a psychological burden of illness. The unnecessary use of medical examinations also indicates a poor prioritisation of society’s resources [5].

Overuse of radiological examinations is a recognised problem within medical overactivity. In Norway, not only has there been a general increase in the use of imaging diagnostics, but there has also been a great geographical variance [6]. Increasing the use of imaging in the assessment of nontraumatic musculoskeletal disorders is particularly challenging [7]. Besides technological development, increased availability and expectation of imaging among patients, are some of the drivers of such development [8]. In 2014, the Norwegian Directorate of Health issued national guidelines for imaging nontraumatic musculoskeletal disorders, intended for primary healthcare [6]. These state that imaging should be considered only if it provides additional clinically important information about the diagnosis or treatment.

A study on barriers to the implementation of the guidelines identified several factors influencing unwarranted imaging, including time pressure, patients’ demands and doctors’ need to exclude uncertainty about the diagnosis and fear of misdiagnosing a serious disease [9]. The finding of such ‘doctors precautions’ is supported by other studies [10] and has been pointed out as one reason for overinvestigation [11].

In Norway, the General Practitioner Scheme ensures all residents a regular general practitioner (RGP). Along with a personal responsibility for patients, GPs play a gatekeeper role that involves assessing which patients should be referred for imaging and specialist health services. This role can be difficult for GPs to maintain, and fear of conflict can make it difficult for them to resist requests from patients for referrals that are not medically justified [12]. Meanwhile, as gatekeepers, GPs are in a unique position to prevent medical overuse by guiding patients to the right level of care through purposeful communication.

Research in this area is scarce, and there is a need for increased knowledge about GPs strategies. This study aimed to explore GPs’ strategies in encounters with patients with expectations for imaging that are not medically indicated according to current guidelines, as well as how patients experienced these strategies.

## Materials and methods

### Design, setting and data collection

**Figure 1.**
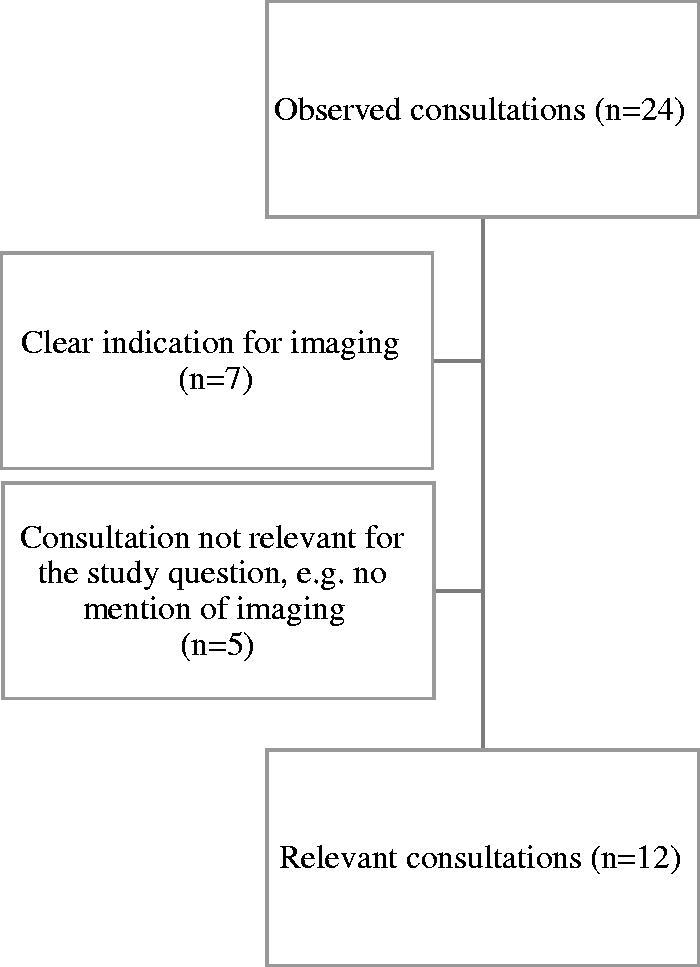
Selection of relevant consultations.

We chose a qualitative study design using both observations and interviews. We conducted convenience and purposive sampling, selecting participants based on accessibility but also aiming for variation in geography, age, gender and experience. We recruited nine GPs (four women and five men) from two different GP offices in two Norwegian urban areas. The study was conducted in February 2020. One of the first authors (KW or MN) was present as a participating observer in their role as a medical student in 24 consultations where the patient was expected to propose a referral to imaging. Consultations for observation were selected based on the stated cause of contact in the GPs’ schedule or by the GPs themselves. Short, focused interviews with patients and doctors were separately conducted after the consultations. An observation sheet for field notes, as well as semistructured interview guides, were used. The observation was focused on describing GPs’ strategies and doctor–patient interactions. In the interviews, we were interested in how the consultation was experienced by both parties. The GP interviews also included questions about preferred strategies to avoid overuse. In total, 12 of the consultations performed by six GPs were considered particularly relevant, varied and well suited for the purposes of the study [13] and were therefore selected for a more thorough analysis (Figure 1). Characteristics of GPs and patients are presented in Table 1.

**Table 1.** Characteristics of the participants, patients (1–12) with their corresponding/regular GP (G, I, A, C, D, E) and D/P-relationship = duration of doctor-patient relationship, M = male, F = female.

Patients				**D/P relationship** **(years)**	Doctors				
*Nr*	*Gender*	*Age (years)*	*Disease, conditions*		*Nr*	*Gender*	*Age (years)*	*Listed patients*	*Experience As GP (years)*
1	F	65–70	Elbow trauma	19–20	G	M	45–50	1300	20
2	F	55–60	Numb thumb	20					
3	F	50–55	Knee pain	27	I	F	60–65	1350	31
4	F	30–35	Hand injury	27					
5	F	40–45	Request mammography	14	A	F	55–60	1300	28
6	M	55–60	Neck pain	27					
7	M	45–50	Shoulder pain	17	C	M	50–55	1300	23
8	F	40–45	Back pain	18					
9	M	45–50	Shoulder pain	5					
10	M	35–40	Knee + thumb trauma	1	D	M	50–55	1100	14
11	M	50–55	Back pain	2	E	F	40–45	1100	2
12	F	25–30	Knee trauma	2					

All GPs except one were specialists in family medicine with an average work experience of 19 years. Three of the six GPs were women, and their average age was 50 years. The average list length was 1,250 patients. Three of the GPs had additional experience in dealing with orthopaedic issues, while three others had experience teaching medical student with a focus on physician–patient communication. Six of the 12 patients were women, and their average age was 49 years. Three of the patients were healthcare workers. They presented with different medical issues. The average duration of the doctor–patient relationship was 15 years.

### Analysis

The data consisted of observational field notes from 12 selected consultations by six GPs, as well as notes from the corresponding GP and patient interviews. In addition, interview data from all nine GPs describing commonly used strategies to avoid overuse were included.

The analysis followed an interpretative phenomenological approach (IPA), which relies on the theoretical framework of phenomenology, focusing on individual experiences as a valid source of knowledge [14]. The analysis involved performing repeated readings to get an overall impression, identifying emerging themes and looking for patterns and identifying superordinate themes. This procedure was repeated for each case, and in the end, patterns across cases were identified. All authors read the data material separately and identified preliminary themes, which were later compared. Different interpretations were discussed and clarified before reaching an agreement on the final overarching themes. Observational data were analysed with a focus on categorising different GP strategies. Categories that fit with well-known GP working methods were identified first, and then some new categories were formulated. Overlapping categories were compared, and possible mergers were considered. In the analysis of the interviews, we focused on the GPs’ and patients’ experiences with different strategies and their perceptions of the consultations. We then read all data related to each patient case (field notes, interviews) longitudinally to compare the experiences of the different parties with the consultations. Finally, we reviewed the GPs’ reports on the strategies they commonly used and then compared these with the strategies we observed ourselves.

### Ethics

The study protocol was evaluated by the Regional Committee for Medical and Health Research Ethics (REC) but did not require such approval (reference 2019/723). The study protocol was approved by the Norwegian Center for Research Data (NSD) (reference 159367). All participants provided written informed consent.

## Results

We identified a total of five categories of GP strategies to avoid unwarranted use of diagnostic imaging, as highlighted in Table 2. We also found that GPs usually combined several strategies rather than just using one. Below we present these different categories with some illustrative examples.

**Table 2.** Observed categories of GP strategies.

Observed categories of GP strategies
1) Wait and see – or suggest an alternative 2) The art of rejection 3) Seek support from a professional authority 4) Partnership and shared decision making 5) Reassurance, normalisation and recognition

### GP strategies to avoid medical overuse of diagnostic imaging

#### To wait and see – or suggest an alternative

A common strategy was to ask the patient to give the symptoms time to pass without any interactions. This strategy was often combined with a safety plan for what to do if the symptoms did not resolve. The GPs described this approach during the interviews; for instance, GP B discussed one of his usual strategies:

*I say, for instance, that we should wait a week and see if there is a clear deterioration, and that we can refer later if it is not better. Patients usually accept this.*

This strategy was closely related to the one where GPs chose to ‘suggest an alternative’ to the imaging that the patient requested and sometimes also involved elements of ‘wait and see’. For instance, several of the GPs prescribed NSAIDs or analgesics instead of referrals to the diagnostic imaging of musculoskeletal disorders. One of the GPs’ offices had a good collaboration with a physiotherapist that used ultrasound examination as part of his treatment of musculoskeletal disorders, and we observed that in some cases they recommended referrals to this physiotherapist instead of proceeding with the requested imaging. This strategy was also confirmed by the GPs in the interviews, as illustrated by the following statement by GP A:

*I often refer to a specialised physiotherapist who does ultrasound examinations and argue that it is a much better examination than MRI. It does not cost society as much and it is actually better.*

#### The art of rejection

In several consultations, we observed that the GPs communicated a clear rejection of the patient’s request for imaging referral. This was done in several ways. Some GPs refused without giving the patient any reason or opportunity to present any counterarguments. Others discussed with the patient and spent more time explaining why imaging was not indicated, such as why an X-ray would not change the treatment. A third variant was to ‘get ahead of the patient’ by stating clearly that imaging was not indicated even though the patient had not (yet) made any suggestions. This strategy was also mentioned in the GP interviews as exemplified by the statement of GP H:

*In general, if they request a specific examination, I give them an explanation of the relevant findings. I explain what is known and that getting, e.g. an MRI is not worth waiting for. Any findings on the MRI scan, will not have consequences for the treatment.*

In several cases, the GPs explained their diagnostic approach for the patient, often thinking aloud during the consultation. We observed that when the GPs used this strategy, they empathised with the patients by sharing the reasoning behind their conclusions to a larger extent. The GPs used the medical history and findings from the clinical examination to explain in a pedagogical way why imaging was not indicated. This explanatory strategy was also confirmed and described by the GPs during the interviews. For example, GP I described one of her commonly used strategies as follows:

*I usually talk about the patient’s problem and explain why the imaging is not necessary by pointing out things that can be seen in the clinical examination. I say, for example, ‘Now you have good mobility, so I don’t think there is anything wrong with the skeleton’, or point out that the patient doesn’t have radiating pain explaining why it was unnecessary to refer to imaging*.

#### To seek support from a professional authority

We observed that the GPs sometimes consulted a more experienced colleague at the same office or a hospital specialist before deciding. This strategy was also mentioned in one of the GP interviews as a method for dealing with uncertainty. As GP A explained,

*I am often a little insecure, and I feel that I consult with colleagues more often than my colleagues at the GP office. I want to do the right thing.*

We also observed that some of the GPs referred to professional authorities when arguing against referrals to imaging. For instance, they mentioned national guidelines and recommendations or referred to other medical specialists.

#### Partnership and shared decision-making

We observed that most GPs emphasised cooperation and partnership with the patients and were usually open for codetermination. This emerged both from the way the GPs formulated themselves (by saying ‘we’ instead of ‘I’) and, in some cases, from letting the patients choose for themselves after informing them of their medical opinion. There was a tendency for more codetermination in cases where the GPs were unsure of the adequate medical level of examinations, if they were afraid to overlook serious illness or if the patients had a health professional background or private health insurance. An example emerged in the interview with GP I, who decided not to refer the patient to imaging:

*He accepted it when I said that I thought it was not necessary to take an X-ray, you know, someone has to make that decision. If he had said, ‘But I would like an X-ray’, I might have sent him there. It is an interaction with the patient.*

#### Reassurance, normalisation and recognition

We observed that the GPs were concerned with taking patients seriously but at the same time normalised certain ailments and reassured patients when their symptoms did not represent serious illness. An illustrative example emerged in the consultation between GP G and patient 2, a woman in her fifties with arm numbness who requested an MRI. The GP then pointed to aging as a common and normal phenomenon in her age group:

*There is a bit of wear and tear with age. If you take an MRI of all healthy 50-year-olds, you will find some changes.*

The same GP reported in the interview that one of his commonly used strategies was to positively respond to all patients’ requests for imaging (recognition of the patient) even though he does not think that the examination is indicated. He usually responds to the patient’s request by saying, ‘That was a really good question!’ before going on to explain why the examination is not necessary.

### The patient perspective and aspects of the doctor–patient relationship

One of the study’s aims was to explore how patients experienced GPs’ strategies and how they affect the doctor–patient relationship. Except for a few improvement suggestions for some parts of the consultations, the patients consistently expressed satisfaction with the doctor’s decisions even where their referral requests were rejected. This emerged both as spontaneous comments from the patients in the interviews and as answers to our questions. The patients expressed that they felt taken seriously, that they were confident in the doctor’s decision and that they could make new contact if the situation changed. We have not been able to find any obvious connection between the GP’s choice of strategy/communication style and patient satisfaction. However, during the analysis, it became evident that aspects related to the doctor–patient relationship had an impact on both GP’s and patient’s experience of the consultation and the decisions that were made. This emerged as we read the material longitudinally and compiled observational data and the GP and patient interviews. We identified three relevant aspects of the doctor–patient relationship: (1) the patient’s confidence in their GP over time, (2) the GPs’ knowledge of the patient’s medical history and (3) the GPs’ knowledge of the patient’s background, personality and behaviour. This finding was evident in most consultations.

Table 3 illustrates an example where all three aspects of the abovementioned doctor–patient relationship are significant. In this case, the GP’s knowledge of the patient’s medical history and personality seems decisive for the way she handles the situation.

**Table 3.** Excerpt from observational field notes from the consultation dialog between patient 6 and GP A and from the following interview with the patient and the GP after the consultation.

**An illustrative case – summary of context from field notes:** *Patient 6, a 55- to 60-year-old male, has had GP A as his regular GP for 27 years. The patient does not speak Norwegian fluently. He has now shown up and demanded to see his GP without any appointment or good reason to the secretary. GP A takes the patient into her office and finds out that the patient is there to demand a new MRI due to persistent head and neck pain after an injury. This context gives the consultation a slightly skewed start. The interaction between GP A and patient 6 is, however, characterised by familiarity. They obviously know each other well. They laugh and communicate with humour during the consultation. The patient appears uneasy, worried and a little difficult to convince in the beginning.*		
Dialogue in the consultation	Patient 6 interview	GP A interview
P 6: ‘I need an MRI of my neck!’	**Why did you choose to see a doctor?**	**How well do you feel you know the patient?**
GP A: ‘In my opinion, we know why it hurts. What we don’t know is how to get rid of the pain. We know that a joint in your shoulder has been dislocated. Those who work with this a lot say that this particular joint is the worst to get dislocated, so it is no wonder that you are in pain’.	Need help. I am worried and in despair. Have known the doctor for a long time. Feel safe. **What did the doctor do?**	Very well! **To what extent do you feel that you reached a common understanding with the patient regarding further management of the problem?**
P 6: ‘But now it hurts further up in the neck and head!’ GP A: ‘The pain probably spreads in the muscles’.	Did not refer to an MRI. **What do you think about the doctor’s decision?**	I’m a little unsure whether we agreed or not. I felt that I made the decision, but he also said that he was reassured by what I said, so I thought it was okay.
P 6: ‘I have had physiotherapy, cracking and massages and I am still in pain!’	Satisfied. Not unhappy/dissatisfied that I did not get an MRI.	**Do you have any other thoughts about the consultation?**
*[Pain killers are discussed]* GP A: ‘Do you think you want surgery?’ P 6: ‘No’. GP A: ‘Then there is no point in doing another MRI scan. You have already done that. We won’t find anything new. A new MRI of your neck won’t change the further treatment. The fact that physiotherapy doesn’t help does not mean that there is something wrong with your neck other than what we already know’.	**If you were worried, are you still worried?** The pain is still there, I was scared, sceptical to what is there. Honestly, afraid that it is cancer when it doesn’t pass. Now I’m less worried about cancer. They know what’s wrong. I do not want surgery. I was calmed, got good answers.	I was annoyed that he said something else than what he really came for to get in at once. At the same time, I know him very well and know that he is very worried. In addition, I rarely get very upset as I am used to people being a bit rude.
*[The patient sits back in the chair and relaxes]* P 6: ‘I am calming down now’.		

## Discussion

### Main findings

In this study, we found that the GPs used a broad range of strategies to avoid medical imaging and usually combined several strategies rather than using just one. The patients appeared generally satisfied regardless of the strategy used by the GP and even where their referral requests were rejected. Additionally, we found that factors related to a long-term doctor–patient relationship seemed to affect both the communication and experience of the consultation for both parties. Factors that appeared important were the patient’s trust in the GP and the GP’s knowledge of the patient’s medical history and their background, personality and behaviour.

### Strengths and limitations

The major strength of our study is that real practice was observed, and triangulation of data provided insights into a complex phenomenon from different angles. The GP participants were from two different parts of the country, and the group was heterogeneous in terms of age, gender and experience, which might indicate that the findings may be transferable to other GPs. However, since only two GP offices were included, and three out of six observed GPs had university affiliation, they are not necessarily representative for regular GPs. The participants were informed of the purpose of the study in advance, which may have increased the GPs’ awareness and stimulated them to act more ‘correctly’ than they would otherwise have done. All GPs worked in urban areas, and the majority of doctor–patient relationships was long-lasting, meaning that the results may be less transferable to rural GPs and/or shorter doctor–patient relationships. Several of the patients sought help for minor orthopaedic complaints. The results might be less transferable to patients with other symptom presentations. All patients were assured regarding confidentiality, but some might have been reluctant to express criticism of the GP’s handling. As health personnel, we have the advantage of being familiar with the field being observed. All observations affect those who are observed. To minimise the impact, the observer was positioned away from the doctor and the patient so that they could talk as undisturbed as possible. Two of the observed GPs were known to us from before, while the rest were unknown. We were aware of this during the analysis but have not identified any obvious differences in the data material.

### What is known from before – and what does our study add?

Several of the identified strategies are recognisable as well-known working methods and consultation skills in general practice [15]. This implies that the GPs used their regular toolbox in situations where they wanted to prevent unwarranted referrals to imaging. One of the most common strategies was to ask the patient ‘to wait and see’, often combined with a safety plan for what the patient should do if the symptoms did not resolve. This strategy is similar to ‘watchful waiting’, often referred to as part of the ‘test of time’ diagnostic strategy in general practice [16]. Approximately 50% of health problems in the primary health service are due to transient or harmless conditions, and seeing the symptoms develop over time can contribute to diagnostic clarification. This is considered a safe method when done in combination with a safety plan, often referred to as ‘safety netting’, another key principle in general practice [17]. However, it is also important to keep in mind that this strategy might have some disadvantages, such as delayed diagnostics and treatment.

‘To wait and see’ was closely associated and sometimes coincided with ‘suggest of an alternative’, which could involve prescribing medications instead of referrals to diagnostic imaging. This strategy resembles another common diagnostic strategy in general practice, ‘test of treatment’, where a diagnosis can be confirmed or refuted by assessing the effect of a treatment [16]. Another alternative that the GPs mentioned was referral to a physiotherapist who used ultrasound to facilitate the treatment. The GPs argued that this was less expensive and contributed to a better outcome. The use of such ‘point-of-care ultrasound’ is currently being implemented in primary care and is in line with the national guideline that mentions increased use of ultrasound as a measure to reduce unnecessary referrals to MRI [6]. Although this strategy does not lead to less diagnostic imaging, it is much more cost-effective.

Not surprisingly, we also found that several of the strategies were closely related to key consultation skills in the patient-centred approach, the preferred communication method in general practice. The ‘partnership and shared decision-making’ and ‘reassurance, normalization and recognition’ strategies are recognisable consultation skills from these models [18]. By acknowledging the patient’s complaints, concerns or needs, doctors show that they take patients seriously and also have the opportunity to express empathy and support, which are important aspects of the patient-centred approach [18].

Managing uncertainty is considered an essential skill in general practice, as it is common for patients to present unclear problems that do not always have an obvious diagnosis. The strategy category ‘to seek support from a professional authority’ includes well-known advice that has been presented as a method to address insecurity in general practice [15]. Uncertainty among doctors has been suggested to be a driving force for medical overuse in primary healthcare services, and reducing it can be seen as a form of quaternary prevention (to protect individuals from overmedicalization) [19]. Seeking support from national guidelines might also prevent overuse by ensuring the right level of care and the prioritisation of resources [6].

In several of the consultations, we observed that the GPs rejected the patients’ requests for referrals to imaging. This was done in several ways, ranging from a clear, direct rejection without any explanation to more indirectly trying to ‘get ahead of the patient’. An American study describes similar findings in which physicians justified their refusals by referring to the lack of indication for imaging and treatment consequences, the cost of the tests and the disadvantages of the examinations [20]. It is considered good patient-centred practice to directly explore patients’ expectations in the consultation, but according to a study from South Wales, GPs, in fear of confrontation, would rather use indirect methods ‘to convince’ the patient [21]. An American study exploring primary-care physicians’ strategies for communicating request denials reports that patients were more satisfied if their perspectives were discussed even though their expectations were not met [22].

The overall impression from our study was that most patients seemed satisfied despite experiencing rejection but that this might be due to factors associated with a long-term doctor–patient relationship. This is well in accordance with the patient-centred approach in which trust in the doctor and building relationships constitute the very foundation of the patient-centred model [18].

Several of our findings are consistent with those of previous research. A recent study by Opdal *et al.* [11] identifies four major strategies for avoiding overtesting. Some of these strategies fit well with our findings, including saying no to patients and negotiating with them. However, while the GPs in Opdal’s study expressed frustration with having to compromise with patients, the GPs in our study appeared confident that they had proposed adequate medical alternatives. The strategy that was deemed most promising in Opdal’s study, ‘to share medical uncertainty and fallibility’, resembles the ‘partnership and shared decision-making’ strategy in our study. The concept of shared decision-making means that doctor and patient make common decisions based on knowledge, experience and the patient’s preference and are in accordance with a desired development, where the patients’ right to codetermination is emphasised. In our study, the GPs did not explicitly discuss the patient’s symptoms in a broader perspective, for example, in relation to psychosocial factors as described by Opdal. However, one might argue that the patient-centred communication technique ‘reassurance, normalization and recognition’ is characterised by the same importance placed on empathy and awareness of the patient’s perspective.

In a German study by Alber *et al.*, GPs identified several drivers of medical overuse and proposed various strategies for reducing it, including a wait-and-see approach and a trustful doctor–patient relationship based on shared decision-making [19]. This is consistent with our findings. ‘Watchful waiting’ was one of the most commonly used strategies in our study, often in combination with ‘reassurance, normalization and recognition’ or suggesting an alternative. In cases of rejection, most GPs were concerned with justifying their refusal to the patient.

These strategies are also in accordance with the findings of an American study by May *et al.* [23], which examined consultations between primary care physicians and standardised patients requesting referral to imaging examinations of low diagnostic value. The study included an intervention to increase doctors’ communication skills, with emphasis on patient-centred communication. This was combined with watchful waiting. Although a general increase in patient-centred approach did not seem to affect referral rates, the waiting strategy was associated with a 39% lower probability of referral [23]. Another American study has shown that patient-centred communication is associated with not only lower costs of diagnostic testing but also longer visits [24]. A Dutch study from 2009 examined the extent to which GPs’ test-ordering strategy towards patients with medically unexplained symptoms affected patients’ satisfaction with and anxiety after the consultation [25]. The study failed to demonstrate any differences between immediately ordering tests or watchfully waiting.

Our study suggests that the doctor–patient relationship had an impact on both GP’s and patient’s experiences of the consultation and the decisions that were made. Several other studies suggest similar effects. A trusting doctor-patient relationship has been highlighted as one of the most promising “quaternary prevention measures” measures in primary care [19]. Familiarity with a patient makes the interpretation of nonverbal communication easier, helps the patient bring up sensitive topics, promotes compliance and seems to make consultations more effective [18]. However, attributing such a large and extensive task to GPs can be difficult given the increasing workload in general practice [26].

The importance of continuity in care is well documented. Studies have shown that continuity in primary care is associated with reduced referrals to outpatient clinics and hospitalisation and that sudden discontinuity in practice increases the risk of acute hospital admissions [27–29]. Continuity also enables person-focused care, where the GP accumulates knowledge of the patient as a person over time, which in turn provides the possibility for a better recognition of patients’ health complaints and facilitates tailored care [30].

## Implications

The GPs participating in this study demonstrated both awareness and a wide range of specific strategies to prevent unwarranted referrals to diagnostic imaging. Continuity in the doctor– patient relationship and good knowledge of the patient seemed essential. Previous research has suggested that GPs are assigned a vital role in quaternary prevention in primary care. The results from our study support this view. Even though more research is needed to examine the actual effects of GP strategies, the findings can be useful for doctors who want to expand their repertoire of strategies when dealing with similar issues. We consider it important that doctors generally develop strategies that involve the patient in reasoning around – and a common understanding of the importance of – avoiding referrals to unnecessary imaging. In addition, we envisage that these strategies can be used for teaching purposes.
